# Supplementary material for: Dietary Protein Quality Affects the Interplay between Gut Microbiota and Host Performance in Nile Tilapia
Source: Animals (Basel). 2024 Feb 24;14(5):714. doi: 10.3390/ani14050714 (PMC10930938; doi:10.3390/ani14050714)
Supplement: Supplementary file 1 [file animals-14-00714-s001.zip › animals-2731219-supplementary.pdf]

Supplementary material

Figures:

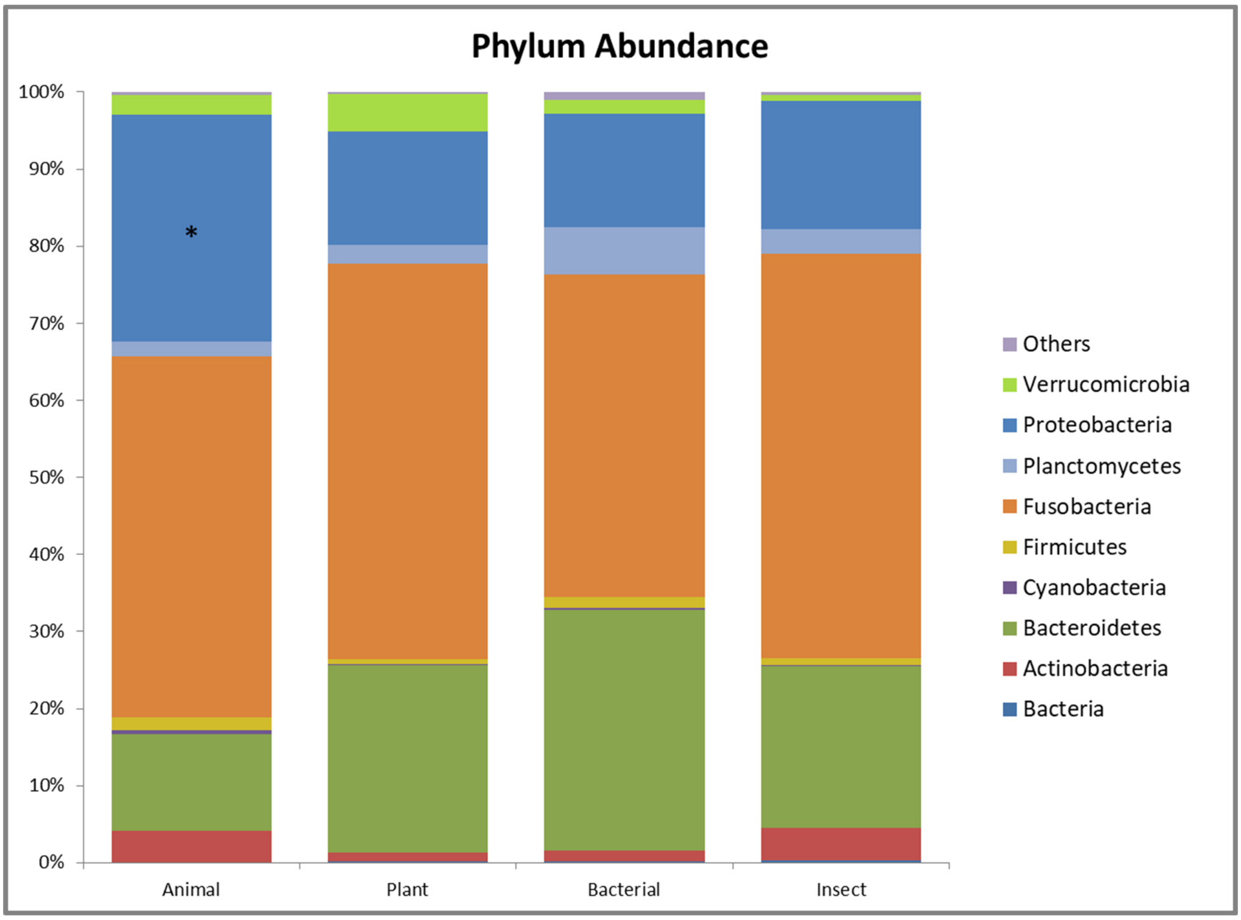

Figure S1: Percentage of abundance at phylum and genus level for tilapia fed diets containing different protein sources: Animal, insect, Plant and Bacterial. Legend represents the most abundant phyla for each treatment.

Tables:

Table S1: Absolute values for amino acid intake per treatment.

Amino Acids	Intake (mg/fish/day)			
	ANIMAL	PLANT	BACTERIAL	INSECT
Arg	36.27	33.43	36.18	33.65
His	12.77	11.89	10.95	10.56
Ile	18.30	19.16	18.94	18.04
Leu	37.83	41.96	38.49	41.02
Lys	26.42	24.71	27.45	29.01
Thr	18.79	17.74	27.12	19.31
Trp	4.09	4.15	4.45	4.13
Val	21.37	21.57	22.08	22.79
Met	10.95	11.24	12.91	11.59
Cys	6.18	5.86	5.25	6.64
Phe	24.73	26.13	23.25	23.63

Tyr	21.93	24.69	21.08	21.68
Asx	32.27	32.57	34.14	33.82
Glx	64.61	78.88	67.94	71.75
Ala	24.81	23.81	26.07	25.53
Gly	33.97	25.80	30.40	30.87
Pro	30.58	33.08	28.35	31.74
Ser	20.09	21.59	19.81	24.07

Table S2: Whole body composition of fish under the dietary treatments.

Diet	Fresh matter				
	**Moisture (%)**	**Ash (%)**	**Protein (%)**	**Fat (%)**	**Energy (kJ/g)**
Initial	76.28	4.26	14.99	4.07	4.88
ANIMAL	73.93±0.42	3.12±0.18	14.80±0.34	6.99±0.23	6.07±0.07
PLANT	73.90±0.85	3.16±0.03	14.70±0.63	7.18±0.12	6.12±0.18
BACTERIAL	73.99±0.46	3.61±0.35	14.67±0.44	6.61±0.23	5.94±0.12
INSECT	74.53±0.63	3.16±0.19	14.60±0.64	6.44±0.13	5.82±0.17

Table S3: DADA2 table output

sample-id	Treatments	input	filtered	% of input passed filter	denoised	merged	% of input merged	non-chimeric	% of input non-chimeric
S20-31-1-V1V2	Animal	65361	43059	65.88	42695	41815	63.98	28473	43.56
S20-31-10-V1V2	Plant	74653	51726	69.29	51062	49332	66.08	40537	54.3
S20-31-11-V1V2	Plant	73776	53734	72.83	53000	50676	68.69	36095	48.93
S20-31-12-V1V2	Plant	84180	59461	70.64	58528	55781	66.26	40487	48.1
S20-31-13-V1V2	Animal	88619	60274	68.01	59193	56472	63.72	43155	48.7
S20-31-14-V1V2	Animal	67321	47629	70.75	47233	46029	68.37	30943	45.96
S20-31-15-V1V2	Animal	63888	32119	50.27	31854	31338	49.05	21373	33.45
S20-31-16-V1V2	Bacterial	12371	81867	66.17	80707	77695	62.8	60910	49.23
S20-31-17-V1V2	Bacterial	84987	62793	73.89	61964	57777	67.98	34241	40.29
S20-31-18-V1V2	Bacterial	87991	43759	49.73	43345	42244	48.01	29282	33.28
S20-31-19-V1V2	Insect	76437	55109	72.1	54322	50400	65.94	33902	44.35
S20-31-2-V1V2	Animal	58065	31359	54.01	30898	29589	50.96	20574	35.43
S20-31-20-V1V2	Insect	10713	31671	29.56	31371	30959	28.9	22470	20.97
S20-31-21-V1V2	Insect	56981	37977	66.65	37613	36502	64.06	29717	52.15
S20-31-22-V1V2	Plant	63715	44995	70.62	44456	42967	67.44	31679	49.72
S20-31-23-V1V2	Plant	40893	25787	63.06	25482	24614	60.19	19473	47.62
S20-31-24-V1V2	Plant	99616	74154	74.44	73336	68780	69.05	42222	42.38
S20-31-25-V1V2	Animal	62697	46641	74.39	45609	43345	69.13	26409	42.12
S20-31-26-V1V2	Animal	52954	37693	71.18	36883	35157	66.39	25936	48.98
S20-31-27-V1V2	Animal	51596	31574	61.19	31139	30160	58.45	26060	50.51
S20-31-28-V1V2	Bacterial	66053	49000	74.18	48345	45724	69.22	27647	41.86
S20-31-29-V1V2	Bacterial	61913	39740	64.19	39189	37920	61.25	25963	41.93
S20-31-3-V1V2	Animal	55504	30152	54.32	29935	29369	52.91	19833	35.73
S20-31-30-V1V2	Bacterial	61983	45692	73.72	45026	43055	69.46	28294	45.65
S20-31-31-V1V2	Insect	49172	34598	70.36	34148	32022	65.12	18318	37.25
S20-31-32-V1V2	Insect	89435	64527	72.15	63848	61559	68.83	41641	46.56

S20-31-33-V1V2	Insect	65507	49563	75.66	48990	47076	71.86	30249	46.18
S20-31-34-V1V2	Plant	65767	49341	75.02	48651	46343	70.47	28455	43.27
S20-31-35-V1V2	Plant	52733	35874	68.03	35456	34081	64.63	28858	54.72
S20-31-36-V1V2	Plant	76281	51974	68.13	51114	48694	63.84	33877	44.41
S20-31-4-V1V2	Bacterial	63161	45839	72.57	45549	43250	68.48	31674	50.15
S20-31-5-V1V2	Bacterial	53890	40868	75.84	40750	39859	73.96	31914	59.22
S20-31-6-V1V2	Bacterial	65243	44027	67.48	43762	42751	65.53	24744	37.93
S20-31-7-V1V2	Insect	45853	32869	71.68	32698	31604	68.92	26115	56.95
S20-31-8-V1V2	Insect	110255	74680	67.73	73368	70675	64.1	49777	45.15
S20-31-9-V1V2	Insect	50559	38391	75.93	38024	36784	72.75	26320	52.06

Table S4: Permanova values for weighted and unweighted Unifrac.

Group 1	Group 2	Sample size	Permutations	pseudo-F	p-value	q-value
**Unweighted-Unifrac**						
Animal	Insect	18	999	1.127	0.272	0.3432
	Bacterial	18	999	1.283	0.152	0.3432
	Plant	18	999	1.119	0.286	0.3432
Insect	Bacterial	18	999	0.940	0.485	0.485
	Plant	18	999	1.432	0.098	0.3432
Bacterial	Plant	18	999	1.241	0.175	0.3432
**Weighted-Unifrac**						
Animal	Insect	18	999	0.838	0.461	0.564
	Bacterial	18	999	2.124	0.077	0.231
	Plant	18	999	2.535	0.051	0.231
Insect	Bacterial	18	999	0.728	0.556	0.564
	Plant	18	999	0.720	0.564	0.564
Bacterial	Plant	18	999	0.825	0.472	0.564

Table S5: Abundance of the predicted pathways that evidenced correlation with amino acid intake in the three diets when compared with ANIMAL.

**ANIMAL vs PLANT**			
**Pathway**	**Superclass**	**ANIMAL**	**PLANT**
4-aminobutanoate degradation V	Amine and Polyamine Degradation	0,12	0,07
creatinine degradation II	Amine and Polyamine Degradation	0,00	0,00
aromatic biogenic amine degradation (bacteria)	Amine and Polyamine Degradation	0,11	0,05
L-arginine biosynthesis	Amino Acid Biosynthesis	0,42	0,35
L-glutamate and L-glutamine biosynthesis	Amino Acid Biosynthesis	0,12	0,12
L-isoleucine biosynthesis	Amino Acid Biosynthesis	0,33	0,34
L-lysine biosynthesis I	Amino Acid Biosynthesis	0,69	0,77
L-histidine degradation I	Amino Acid Degradation	0,46	0,45
L-leucine degradation I	Amino Acid Degradation	0,14	0,09
L-lysine fermentation to acetate and butanoate	Amino Acid Degradation	0,43	0,50
pyruvate fermentation to butanoate	Fermentation	0,12	0,16
pyruvate fermentation to propanoate I	Fermentation	0,32	0,36
mixed acid fermentation	Fermentation	0,41	0,49
**ANIMAL vs MICROBIAL**			
**Pathway**	**Superclass**	**ANIMAL**	**BACTERIAL**
L-histidine biosynthesis	Amino Acid Biosynthesis	0,31	0,39
L-isoleucine biosynthesis I (from threonine)	Amino Acid Biosynthesis	0,77	0,84
L-lysine biosynthesis I	Amino Acid Biosynthesis	0,60	0,62
L-methionine biosynthesis I	Amino Acid Biosynthesis	0,23	0,15
L-ornithine biosynthesis	Amino Acid Biosynthesis	0,27	0,36
L-tryptophan biosynthesis	Amino Acid Biosynthesis	0,30	0,41
L-valine biosynthesis	Amino Acid Biosynthesis	0,78	0,84
L-histidine degradation I	Amino Acid Degradation	0,46	0,39
L-arginine degradation II (AST pathway)	Amino Acid Degradation	0,02	0,02
glycerol degradation to butanol	Fermentation	0,11	0,06
homolactic fermentation	Fermentation	0,48	0,57
heterolactic fermentation	Fermentation	0,09	0,05
4-aminobutanoate degradation V	Amine and Polyamine Degradation	0,12	0,06
aromatic biogenic amine degradation (bacteria)	Amine and Polyamine Degradation	0,11	0,04
allantoin degradation IV (anaerobic)	Amine and Polyamine Degradation	0,02	0,01
**ANIMAL vs INSECT**			
**Pathway**	**Superclass**	**ANIMAL**	**INSECT**
superpathway of polyamine biosynthesis II	Amine and Polyamine Biosynthesis	0,16	0,09
4-aminobutanoate degradation V	Amine and Polyamine Degradation	0,12	0,07
allantoin degradation IV (anaerobic)	Amine and Polyamine Degradation	0,02	0,01
L-arginine biosynthesis	Amino Acid Biosynthesis	0,32	0,22
L-isoleucine biosynthesis	Amino Acid Biosynthesis	0,78	0,87
L-lysine biosynthesis	Amino Acid Biosynthesis	0,69	0,79
L-methionine biosynthesis	Amino Acid Biosynthesis	0,23	0,14
L-valine biosynthesis	Amino Acid Biosynthesis	0,78	0,85
L-leucine degradation I	Amino Acid Degradation	0,14	0,08
glycerol degradation to butanol	Fermentation	0,11	0,06
